# Many-faceted rasch calibration of the foot function index-revised short form

**DOI:** 10.1186/s13047-022-00583-y

**Published:** 2022-10-17

**Authors:** Seungho Ryu, Madeline P. Casanova, Jonathan D. Moore, Adam C. Cady, Russell T. Baker

**Affiliations:** 1 WWAMI Medical Education Program, University of Idaho, Moscow, ID USA; 2 Idaho Office of Underserved and Rural Medical Research, University of Idaho, Moscow, ID USA; 3 Cedars-Sinai Kerlan-Jobe Institute, Los Angeles, CA USA; 4 Department of Movement Sciences, University of Idaho, 875 Perimeter Drive, Moscow, ID 83844 USA

**Keywords:** Psychometric evaluation, Patient-Reported Outcomes, Many-Faceted Rasch analysis

## Abstract

**Background:**

The Foot Function Index Revised Short version (FFI-RS) is a foot- and ankle- patient-reported outcome measure (PROM), developed from the Foot Function Index (FFI). Previous studies, estimating item parameters and multidimensional properties, have limitations properly establishing the measurement properties of the FFI-RS. A multi-faceted Rasch analysis with a larger sample would allow for a more robust validation approach to improve the clinical interpretation of the FFI-RS using a multidimensional perspective. Therefore, the purpose of this study was to assess the psychometric properties of the FFI-RS as a PROM of foot function.

**Method:**

A total of 2184 patients with foot pathology who completed the FFI-RS were included in the data. Data were extracted from the cloud-based orthopedic and sports medicine global registry Surgical Outcome System (SOS). The psychometric properties of the FFI-RS were assessed using a many-faceted Rasch analysis that included model-data fit, rating scale function, item-person map (distribution of item difficulty and person ability), and item difficulty of the subscale.

**Results:**

Two misfit items were discovered and deleted; 32-items from the original FFI-RS were retained. The 4-item Likert scale functioned effectively and item difficulty (-0.58 to 1.48), subscale difficulty (-0.58 to 1.15), and person’s foot function (-6.62 to 6.24) had wide distributions.

**Conclusions:**

Many-faceted Rasch analysis revealed the FFI-RS had sound psychometric properties using the many-faceted Ranch analysis and retained 32 of the original items. Clinicians and researchers should consider weaknesses identified with items in the ‘Difficulty” subscale and future work should be conducted to modify or develop items that will more accurately evaluate a wide range of foot function levels.

## Background

The patient’s perspective is an essential parameter in outcome-related research [1, 2]. The focus on outcomes assessment has moved beyond simple evaluation of patient satisfaction to a process designed to assist clinicians and researchers to more accurately capturing how patients perceive the impact of the disease and care on dimensions of health status [3, 4]. Patient-Reported Outcome Measures (PROMs) have been developed to quantify patient perceptions of various health status dimensions (e.g., symptom status, physical function, mental health, social function, overall wellbeing) to help inform research and clinical practice efforts to improve healthcare quality [3–5]. Therefore, PROM utilization can serve as a valuable tool for patient assessment in healthcare [3–7], including following surgical treatment and throughout the continuum of care in orthopedic clinical research [1, 6, 7].

Various foot and ankle-related multidimensional PROMs, such as the Foot Function Index (FFI), revised Foot Function Index (FFI-R), Foot and Ankle Ability Measure (FAAM), Foot and Ankle Disability Index (FADI), Foot and Ankle Outcome Score (FAOS), American Orthopedic Foot & Ankle Society scales (AOFAS), and Ankle Osteoarthritis Scale (AOS) have been commonly used in research and practice [1, 7]. The FFI has been used across various foot-related pathological conditions and has broad appeal to clinicians and research scientists [7–10], which has been supported by its translation into several languages (i.e., Korean [11], Danish [12], Italian [13], Brazil [14], Dutch [8], Spanish [15], German [16], and French [17]). Previous FFI research on the measurement properties of the scale have used Classical Test Theory (CTT) approaches [8, 18] and Rasch analysis [19, 20]. Additional psychometric evaluation identified a need to include additional items (i.e., psychosocial activity and quality of life in foot health) which led to the development of the FFI-R Short version (FFI-RS) [20].

The use of Rasch model psychometric information (e.g., fit statistics, item-person map, rating scale function) helps to provide a more accurate evaluation of the items and measurement of a scale [19–22]. For example, Rasch analysis provides information on how well an item assesses the underlying construct, the possibility of an item's redundancy with other items in the scale, and the acceptability of the response categories. The use of Rasch analysis can help reduce some of the limitations of CTT (e.g., dependency [23], item difficulty) because it produces item statistics independent from the samples and person statistics independent from the items [24], which has led to an increasing use of Rasch analysis in the development and assessment of clinical instruments for healthcare [25]. Traditionally, the Rasch model includes two-facets: item difficulty and person ability; if another facet is added, a many-faceted Rasch model is used [26, 27]. For example, when the five subscales of the FFI-RS (i.e., pain, stiffness, difficulty, activity limitation, and social issues) are incorporated in the modeling, the two-faceted model turns into a three-faceted model (i.e., item, person, and subscale). The analysis also estimates subscale difficulty using a many-faceted Rasch model while simultaneously “controlling for” the subscale difficulty in the estimation of two-facets, item difficulty and person ability parameters.

While the FFI-RS was developed with Rasch analysis, there are limitations with the initial analysis approach and the sample utilized. For example, it may be beneficial to use a larger, more diverse sample which better represents the patient population (e.g., males and females, various age groups) in which the scale is used [28]. Further, it would be valuable to use many-faceted Rasch analysis to simultaneously estimate item difficulty, person ability, and subscales of the FFI-RS to better understand the validity of the FFI-RS from a multi-dimensional perspective. Therefore, the purpose of this study was to evaluate the psychometric properties of the FFI-RS as a patient-reported measure of foot function, using a many-faceted Rasch model with a large and diverse sample of responses from patients who completed the scale.

## Methods

### Data

The data for this study was extracted from the Surgical Outcome System (SOS; Arthrex, Naples, Fl, USA), a cloud-based orthopedic and sports medicine global registry used by registered physicians and facilities. The SOS complies with the Health Insurance Portability and Accountability Act (HIPAA) and has Institutional Review Board (IRB) approval. Patients in the SOS database complete specified PROMs at predetermined time intervals (i.e., baseline [pre-surgery], 3-months post-surgery, 6-months post-surgery, and 12-months post-surgery) and an informed consent as part of the data collection process. Data were downloaded from patients in the United States who had completed the FFI-RS at baseline in this study. The University of Idaho IRB reviewed the protocol for the use of a deidentified SOS dataset and certified the project exempt.

### Foot function index instrument

The FFI instrument assesses an individual’s self-reported foot health based on clinical observations across three constructs: pain (9 items), disability (9 items) and activity limitation (5 items) [28, 29]. The FFI-R was developed to address deficiencies in the FFI related to measuring the psychosocial elements of foot function^30^ and reading level. The reading level was designed at an eighth grade reading level to make it easier for patients to complete, while additional items and constructs were added to allow a wide range of foot function and impairment severity to be measured [30, 31].

The FFI-R exists in two versions: Long (FFI-RL, 67 items) and Short (FFI-RS, 34 items). Both versions are designed to measure 5 subscales: pain, stiffness, difficulty, activity limitations, and social issues. The FFI-RS has a different number of items across the subscales: pain (7 questions), stiffness (7 questions), difficulty (11 questions), activity limitation (3 questions), and social issues (6 questions). Individuals used a 4-point Likert scale to respond, with the option of selecting “Not applicable” to each item. Individuals who selected “Not applicable” for any item were excluded from our analysis. An FFI-RS overall score is calculated by adding the response items, dividing by the maximum total possible for all rated items, and multiplying by 100, with the higher score indicating poorer perception of foot function [18, 29].

### Data analysis

A many-faceted Rasch model [27] was conducted to calibrate the FFI-RS scale with three facets: the item (i.e., difficulty level of knee function), the subscales (i.e., pain, stiffness, difficulty, activity limitation, social issues), and the person (i.e., individually determined level of foot function) using the Rasch Rating Scale Model (FACETS, version 3.71.4). Model–data fit of the FFI-RS was evaluated using the Infit and Outfit statistics. The Infit and Outfit statistics indicate mean squares residuals between observed and expected responses, with Outfit being more sensitive to item or respondent outliers. Both statistics close to 1 designate acceptable fit of the model-data fit; model misfit was concluded if the values were less than 0.5 (little variation) or larger than 1.5 (large variation) [32, 33].

The rating scale function was used to determine if the existing instrument category was appropriate by examining the following criteria [19, 34]: (a) Was there frequency distribution in the observation (such as unimodal, bimodal, and slightly skewed)? (b) Did the average logit measures for each category increase as the category increased? (c) Was the Outfit mean square residual proper for each category (less than 2.0)? (d) Were the category thresholds (i.e., boundaries between rating categories) arranged in order?

A facet map distribution was appraised because it visually represents comparison among the item difficulty, subscale, and person ability estimates on the common scale in logits. The map displayed each participant’s foot function level (difficulty level order in each foot function item), the relative position of a participant’s foot function level for the FFI-RS subscales and items, and the subscale in logits. It is possible to compare item difficulty, FFI-RS subscale, and person ability within the map simultaneously.

Concerning FFI-RS item difficulty and subscale difficulty, the higher the logit score the more challenging the task item. As for foot functional level estimation, the higher the logit score, the lower the person’s foot function level. Separation index and reliability were evaluated for item, subscale, and person. The separation index indicates how evenly the scores and items in the scales were dispersed (separation index > 2.0) [24]. Separation reliability is the capacity to duplicate item or person placements if the same questions were asked in a different sample (reliability values close to 1.00 indicate high confidence for item, subscale, or person reliability) [24].

## Results

### Participant information

The dataset included responses from 2,184 patients (852 males, 1,332 females) suffering from a foot pathology who had completed all items of the FFI-RS at a baseline (i.e., pre-intervention) examination. Patients who had missing demographic data or missing FFI-RS responses were not included in the analysis. Participants ranged from 12 – 90 years (*M* = 47.73 years, *SD* = 16.67 years) of age; participant demographic characteristics are presented in Table 1.

**Table 1:** Demographic characteristics

Characteristic	Mean (SD) / Proportion (n)
Age	47.73 (16.67)
Male (ranged from 12–90 years)	48.44 (16.58)
Female (ranged from 12–90 years)	46.63 (16.77)
Sex	
Male	0.39 (852)
Female	0.61 (1332)

### Model data fit

A total of 2 items had unacceptable Infit and Outfit statistics in the initial analysis: item 26 (Infit value = 2.43 and Outfit value = 3.00), and item 29 (Infit value = 1.76 and Outfit value = 3.11). The final analysis retained 32-items with acceptable fit statistics, Infit statistics ranged from 0.74 – 1.34, while Outfit statistics ranged from 0.70 – 1.39.

### Rating scale functioning

**Fig. 1:**
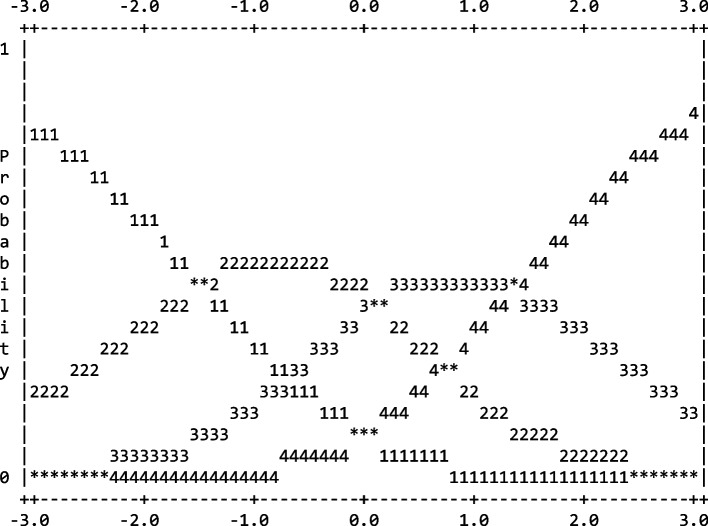
Category probabilities for the 32-item FFI-RS. The logits measures are along the x-axis, and the y-axis represents the probability of each response category across the scale. The figure indicates that plainly defined thresholds for the categories are increasing

The four categorical rating scales of the 32-item FFI-RS are abridged in Table 2. The results support the four categorical rating scales of the FFI worked well. In addition, the category probabilities of the FFI are displayed in Fig. 1.

**Table 2:** Summary of the 32-item FFI-RS rating scale function

*Category Score*	*Counts Used*	*Average Measure*	*Outfit MNSQ*	*Category Thresholds*
1	12,582	-1.74	1.20	None
2	18,312	-0.54	1.00	-1.47
3	17,585	0.58	0.90	0.12
4	14,983	1.89	1.00	1.36

*Average Measure* a mean of logit measures in category, *MNSQ* mean square residuals

### Facet map

**Fig. 2:**
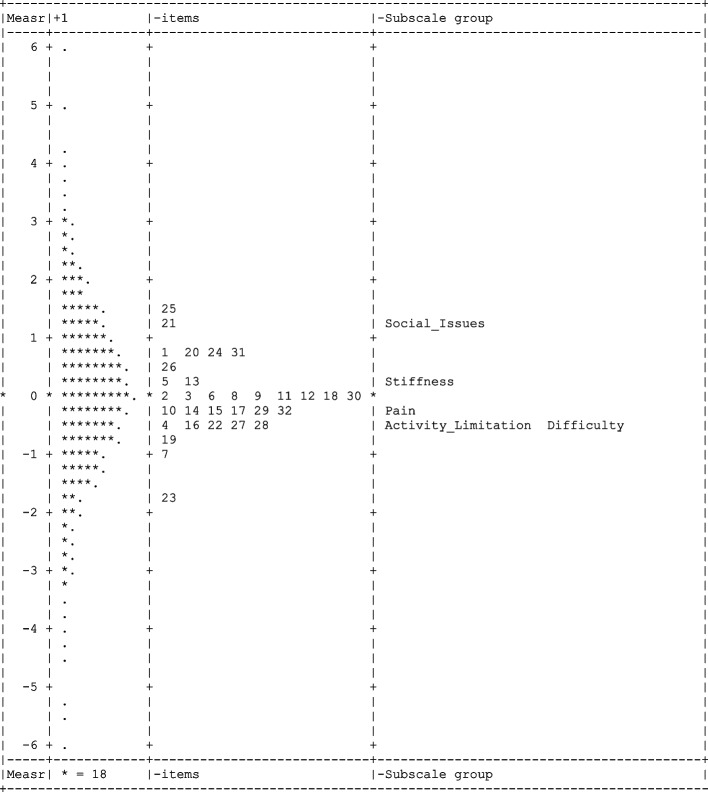
Facet map of the FFI-RS. The distribution displays the logit measures for foot function item difficulty, subscale difficulty, and foot function level. The logit scale on the middle of the map shows the FFI-RS question number and on the right of the map indicates the FFI-RS subscale. The logit scale on the left of the map, indicated by “*” signs, displays the foot function level of persons

The facet map (Fig. 2) shows that participant foot function levels are widely dispensed over the logits scale and the items within the subtests have a reasonable spread. Although FFI-RS items are suitable for most individuals, the items do not cover the content for individuals with the highest (logits > 2) and lowest (logits < -2) levels of foot function. Further, the subscale group (Fig. 2) also denotes that the difficulty levels of the groups are rather limited.

### Item difficulty

Item difficulties, standard errors, and Infit and Outfit statistics for the FFI-RS are reported in Table 3. The FFI item difficulties ranged from -0.58 to 1.48 logits. The most difficult item was question 25 (“Walking with assistive devices”). The least difficult item was question 23 (“Running”). Item separation was 18.36, exhibiting good variability (items were broad on the measurement scale); item separation-reliability was 1.00, indicating a high degree of confidence in repeating item placement within measurement error for another sample.

**Table 3:** Item summary of Rasch calibration in FFI-RS

Item		Subscale	Calibration logits	SE logits	Infit MNSQ	Outfit MNSQ
Q25	Walking with assistive devices?	Difficulty	1.48	.05	1.09	1.05
Q21	Getting out of a chair?	Difficulty	1.29	.03	.95	.89
Q33	Burden of taking medication to control foot pain?	Social Issues	.87	.04	1.34	1.38
Q24	Keeping your balance?	Difficulty	.81	.03	1.19	1.20
Q01	Before you get up in the morning?	Pain	.79	.03	1.23	1.36
Q20	When you carried or lifted objects weighing more than five pounds?	Difficulty	.69	.03	1.08	1.00
Q27	Limit your outdoor activities because of foot problems?	Activity Limitation	.40	.03	1.11	1.16
Q05	When you stood wearing custom shoe inserts?	Pain	.27	.05	.88	.88
Q13	When you walked wearing custom shoe inserts?	Stiffness	.23	.05	.90	.93
Q11	When you stood wearing shoes?	Stiffness	.11	.03	.79	.77
Q03	When you stood wearing shoes?	Pain	.10	.03	.82	.89
Q06	When you walked wearing custom shoe inserts?	Pain	.09	.05	.94	.93
Q08	Before you get up in the morning?	Stiffness	.09	.03	1.09	1.09
Q02	When you first stood without shoes?	Pain	.05	.03	.91	.92
Q32	Difficulty participating in social activities due to footwear?	Social Issues	.04	.03	1.34	1.32
Q09	When you stood without shoes?	Stiffness	-.01	.03	.96	.95
Q18	Descending stairs?	Difficulty	-.07	.03	.75	.71
Q12	When you walked wearing shoes?	Stiffness	-.08	.03	.87	.85
Q17	Climbing stairs?	Difficulty	-.14	.03	.74	.70
Q10	When you walked without shoes?	Stiffness	-.15	.03	.92	.90
Q14	Before you went to sleep at night?	Stiffness	-.19	.03	1.09	1.05
Q31	Limit social activities due to foot problems?	Social Issues	-.24	.03	1.06	1.08
Q34	Concern about limited work around the house?	Social Issues	-.27	.03	.98	.95
Q15	Walking outside on uneven ground?	Difficulty	-.36	.03	.78	.73
Q04	When you walked wearing shoes?	Pain	-.40	.03	.89	.91
Q28	Limit your leisure/sport activities because of foot problems?	Activity Limitation	-.40	.03	1.25	1.28
Q30	Feeling awful because of foot problem?	Social Issues	-.40	.03	1.13	1.39
Q22	Walking fast?	Difficulty	-.58	.03	.86	.78
Q16	Walking four or more blocks?	Difficulty	-.58	.03	.90	.80
Q19	Standing on tip toes?	Difficulty	-.69	.03	1.22	1.09
Q07	At the end of a typical day?	Pain	-.92	.03	1.02	1.09
Q23	Running?	Difficulty	-1.84	.04	1.22	.94

*MNSQ* mean square residuals

### Subscale difficulty

The subscale of the FFI-RS difficulties, standard errors, and Infit and Outfit statistics are reported in Table 4. All subscales had an adequate model-data fit, and all Infit and Outfit statistics were within an acceptable range. The most difficult subscale was “Social Issues” (logits = 1.15), and the least difficult subscale was “Difficulty” (logits =  − 0.58). Subscale separation was 18.66, indicating large variability and subscale separation reliability was 1.00, exhibiting a high degree of confidence in replicating subgroup placement within measurement error for another sample.

**Table 4:** Summary of Rasch calibration of FFI Subscales

*Item*	*Calibration logits*	*SE logits*	*Infit MNSQ*	*Outfit MNSQ*
Social Issues	1.15	0.01	1.15	1.22
Stiffness	0.15	0.01	0.95	0.93
Pain	-0.30	0.01	0.96	1.02
Activity Limitation	-0.42	0.02	1.17	1.22
Difficulty	-0.58	0.01	0.96	0.89

*MNSQ* mean square residuals

### Individual levels

The average foot function level was 0.00 (SD = 1.56). The Individual level of the foot function estimates ranged from –6.62 to 6.24 logits. Person separation was 18.66, demonstrating that a person’s ability widely spread across the measurement scale. The reliability of person separation was 1.00, indicating an excellent confidence level in replicating person placement within measurement error.

## Discussion

The purpose of this study was to evaluate the FFI-RS using the many-faceted Rasch model that included a more extensive and diverse patient sample. Our findings provide further support for sound psychometric properties of the FFI-RS. The FFI-RS item estimates demonstrate appropriate fit and placement for the same metrics. The Rasch analysis resulted in 2 items (Q 26 and 29) being removed from the scale, with the final model retaining 32 items that had acceptable Infit and Outfit statistics.

Our findings did vary from prior research, but this may have been due to the quantity and composition of research participants used in each study, thus resulting in final model solution differences. For example, a previous study [28] had a sample size of 92 patients from a Veterans Administration Hospital podiatry clinic in the Midwest, while our sample included 2,184 participants from an international surgical database. Rasch analysis with smaller sample sizes, like many other statistical analysis procedures, may be less powerful for fit analysis, may be more likely to skew the estimates due to larger standard errors, and may offer less robust estimates [35]. Therefore, it is possible that sample size differences impacted model fit.

Our findings provide novel insight into the response scale for the FFI-RS. The 4-point Likert rating scale met requirements: (a) the responses of distribution of observation across the four categories, with a slightly positive skew; (b) the average logit measures and category thresholds were increased; (c) the Outfits mean square residual were less than 2 for each scale category. Therefore, our findings indicated that the 4-point Likert scale operated effectively in the many-faceted Rasch analysis.

Rasch analysis also has the advantage of individually measuring item difficulty [36]. Item difficulty is related to which items are commonly endorsed (i.e., selected as impaired) by respondents; less difficult items are the ones which are most frequently endorsed (i.e., the item most often endorsed as impaired) by participants and more difficult items are less frequently endorsed. Our findings indicate the subscale “Difficulty” contains both the most and least difficult items for respondents: 1) the most difficult item (i.e., the least commonly endorsed by patients as impaired) was “Walking with assistive devices?”; 2) the least difficult item (i.e., the most commonly endorsed by patients as impaired) was “Running?”. In short, the FFI-RS “Difficulty” subscale contains the item with the lowest likelihood of being selected as impaired by respondents with a foot/ankle pathology, as well as the item (i.e., “Running”) most likely to be reported as impaired by respondents. Further research would be valuable to investigate response patterns based on pathology, symptom or injury severity, and population types (e.g., elite athletes, sedentary patients, different age groups).

Our results also include novel findings with the facet map which displays the items and subscales difficulty distribution of the FFI-RS, the foot function levels distribution of participants, and the relative position among an individual's foot function levels, items, and subscales in the FFI-RS. The end of the facet map has gapping, which means the FFI-RS does not provide a range of content to measure individuals with the highest and lowest levels of foot function. On the logits scale, items measuring persons in the same location may presumably be removed without a major loss of content information. The facet map findings, along with the item difficulty analysis, provide support for further scale modification which could occur with item development or modification to remove or modify redundant FFI-RS items within subscales. The goal would be to develop and evaluate an item pool which fully measures the intended constructs across relevant patient subgroups in future studies.

Our study of the FFI-RS used a larger, more heterogeneous sample (e.g., wide age range [i.e., 12 to 90 years old], larger inclusion of female participants) who sought patient care services for pain or pathology. Thus, our study likely includes a highly generalizable sample of the patient population and novel analysis not previously conducted; however, the use of the dataset from the SOS database and our work does have limitations. First, longitudinal data and a healthy sample of respondents were not included in this study. Future research is needed to assess the longitudinal properties of scale, as well as if the same results are obtained when healthy samples are included to inform if the FFI-RS can be used to differentiate between injured and healthy patients and guide return to activity or patient discharge decisions. Second, item level bias due to age, sex, or ethnicity differences may be a concern; further analysis across other relevant subgroups (e.g., different levels of physical activity or education, injury conditions, interventions) would also be valuable for assessing scale psychometric properties. Therefore, multi-group testing to assess differences across other demographic variables would be valuable.

Our analysis also did not exhaust all psychometric testing that would be valuable. For example, the lack of longitudinal data prevents testing of responsiveness (e.g., minimal clinically important differences) or test–retest reliability, while the lack of available demographic data (e.g., injury type) prevents valuable multi-group analysis from occurring. Lastly, the absence of questions that capture a wider range of foot function is a limitation in our findings. Future research should include various relevant patient populations (e.g., elite athletes, recreational athletes), age groups, pathology/conditions, interventions, and demographic variables (e.g., education levels, ethnicities) to establish the best item pool (i.e., item difficulty range to adequately capture foot function capacity), which would be useful to provide the most valuable information to clinicians while limiting patient response burden across the patient spectrum who could use the scale.

## Conclusions

We utilized the many-faceted Rasch model to provide further insight into the psychometric properties of the FFI-RS. In total, 32 FFI-RS items were identified and retained, evidence for the appropriateness of the Likert scale response structure was found, calibration of the item and subscale difficulties was assessed, and person's ability with a large patient sample was conducted. Many of our findings indicate the FFI-RS has sound psychometric properties of the FFI-RS; however, areas for scale improvement were also noted. Clinicians and researchers should consider weaknesses identified with items in the ‘Difficulty” subscale and future work should be conducted to modify or develop items that will more accurately evaluate a wide range of foot function levels in the patient population while reducing item redundancy to reduce patient response load.

## Data Availability

Data and material are anonymized data on FFI-RS, but raw data is available from the corresponding author upon reasonable request.

## Abbreviations

FFI-RS: Foot Function Index Revised Short version
FFI: Foot Function Index
PROMs: Patient-Reported Outcome Measures
FFI-R: Revised Foot Function Index
FAAM: Foot and Ankle Ability Measure
FADI: Foot and Ankle Disability Index
FAOS: Foot and Ankle Outcome Score
AOFAS: American Orthopedic Foot & Ankle Society scales
AOS: Ankle Osteoarthritis Scale
CTT: Classical Test Theory
SOS: Surgical Outcome System
HIPAA: Health Insurance Portability and Accountability Act
IRB: Institutional Review Board
FFI-RL: Foot Function Index Revised Long version
